# Machine Learning-Based WGCNA Approach for Developing an Immunogenic Cell Death-Related Hub Gene Signature and Identification of AJM1 as a Prognostic Biomarker in Pancreatic Adenocarcinoma

**DOI:** 10.7150/ijms.119960

**Published:** 2025-10-27

**Authors:** Tianyin Ma, Xiangdong Gongye, Cairang Dongzhi, Yibo Chai, Qikun Wang, Ming Tian

**Affiliations:** 1 Department of Hepatobiliary & Pancreatic Surgery, Zhongnan Hospital of Wuhan University, Wuhan, 430071, China.; 2 Department of Chemistry and Molecular Biology, Sahlgrenska Akademin, Göteborg Universitet, Gothenburg, Västra Götalands, Sweden.; 3 Department of Oncology, The Sixth Hospital of Wuhan, Affiliated Hospital of Jianghan University, Wuhan 430072, Hubei, China.

**Keywords:** AJM1, ICD-related hub gene signature, Machine learning, PAAD, WGCNA

## Abstract

**Background & Aims:** Pancreatic adenocarcinoma (PAAD) remains a highly lethal malignancy with limited therapeutic options, primarily due to the absence of reliable prognostic biomarkers. Immunogenic cell death (ICD) plays a pivotal role in anti-tumor immunity and has potential as both a prognostic marker and a predictor of immunotherapy response. This study aimed to identify ICD-related hub genes and establish a robust prognostic gene signature for PAAD using weighted gene co-expression network analysis (WGCNA).

**Methods & Results:** Transcriptomic and clinical data of PAAD patients were obtained from the TCGA and GEO databases. ICD enrichment scores were calculated using single-sample gene set enrichment analysis (ssGSEA), and ICD-associated gene modules were identified through WGCNA. A prognostic ICD-related gene signature was then constructed, and patients were stratified into high- and low-score groups based on the median risk score. Functional enrichment analysis was performed using the Molecular Signatures Database (MsigDB). Correlations between the signature score, immune cell infiltration, and drug sensitivity (IC_50_ values from the GDSC2 database) were further assessed. Among the identified genes, AJM1 emerged as a key prognostic marker, validated in an independent PAAD cohort and through *in vitro* functional assays.

**Conclusion:** This study developed and validated an ICD-related gene signature capable of predicting prognosis and immunotherapy responsiveness in PAAD. The identification and validation of AJM1 highlight its potential role as a prognostic biomarker and a novel contributor to the pathogenesis of PAAD.

## Introduction

Pancreatic adenocarcinoma (PAAD) is among the most lethal malignancies of the digestive system, posing persistent challenges in diagnosis and treatment 1-3. Over half of PAAD patients are diagnosed at an advanced, metastatic stage, resulting in a 5-year survival rate of less than 5% despite advances in surgical techniques and imaging modalities 4,5. At presentation, more than 80% of patients are ineligible for surgical resection, and even those undergoing curative surgery frequently experience local or distant recurrence within two years 6,7. The efficacy of conventional treatments, including radiotherapy, combination chemotherapy, and biotherapy, remains limited, primarily due to the incomplete understanding of PAAD pathogenesis and the lack of reliable biomarkers to guide prognosis and therapy 8-10. Thus, identifying robust molecular biomarkers for diagnosis, treatment selection, and prognostic evaluation remains an urgent clinical priority.

Immunogenic cell death (ICD) has recently emerged as a key mechanism that promotes anti-tumor immune responses, representing a promising avenue for cancer immunotherapy 11-13. Although preclinical studies have highlighted the therapeutic potential of ICD, its clinical relevance in PAAD remains poorly defined. Further research is therefore required to clarify how ICD-related biomarkers can be leveraged to stratify patients based on their likely responsiveness to ICD-based immunotherapies.

To address this gap, we applied a weighted gene co-expression network analysis (WGCNA) approach to identify ICD-associated hub genes in PAAD. Subsequently, Cox proportional hazards and least absolute shrinkage and selection operator (LASSO) regression analyses were conducted to construct an ICD-related prognostic gene signature. This signature was designed to predict patient survival and immunotherapeutic responsiveness while providing mechanistic insights into the potential role of ICD in PAAD progression.

## Materials and Methods

### Data acquisition and processing

#### Training dataset

Clinical and transcriptomic data for PAAD patient samples, normalized in log2(FPKM + 1) format, were retrieved from The Cancer Genome Atlas (TCGA) database using the TCGAbiolinks package in R. Corresponding clinical information for cases included in the training cohort was also collected for subsequent analyses **(Table S1)**.

#### Validation dataset

The GSE57495 dataset (platform: GPL15048), comprising 63 PAAD patient samples, was selected for external validation of the predictive model. Clinical and gene expression data were obtained from the Gene Expression Omnibus (GEO) database, and the corresponding clinical characteristics were summarized in **Table S2**. During data preprocessing, probes without corresponding gene annotations were excluded, and those mapping to multiple genes were removed. For genes represented by multiple probes, the median expression value was used. Since the TCGA (training set) and GEO (validation set) datasets were analyzed independently, batch effect correction was not necessary.

#### Other data

A total of 34 ICD-related genes (listed in **Table S3**) were included in the analyses. The Hallmark gene set was obtained from the Molecular Signatures Database (MSigDB) for pathway enrichment analysis. Statistical significance was defined as p < 0.05, with significance levels denoted as follows: *p* ≤ 0.05 (*), *p* ≤ 0.01 (**), *p* ≤ 0.001 (***), and *p* ≤ 0.0001 (****).

#### Differential expression analysis

Differential expression of ICD-related genes was analyzed by comparing PAAD samples with corresponding control tissues, as well as among patient subgroups stratified by distinct clinical characteristics. The Wilcoxon rank-sum test was used for two-group comparisons, and the Kruskal-Wallis test was employed for multi-group analyses. Single-nucleotide variants (SNVs) of ICD genes in the training cohort were visualized using the maftools package in R. The chromosomal distribution of ICD genes was illustrated with the RCircos package, and copy number variations (CNVs) were plotted using ggplot2.

### Identification and functional enrichment analyses of ICD-associated genes

#### WGCNA of ICD-associated hub genes

Gene expression matrices were used to construct a similarity network using the pickSoftThreshold function of the WGCNA package. The optimal soft-thresholding power (β) was determined to achieve a scale-free topology fit index (R²) > 0.85, ensuring the generation of a biologically meaningful network structure. The ICD enrichment scores for individual samples were calculated using the GSVA package, which employs single-sample gene set enrichment analysis (ssGSEA), and these scores were used to quantify ICD activity for each patient. ICD levels, age, and tumor stage were defined as key phenotypic traits of interest. Samples were subsequently subjected to hierarchical clustering, and gene co-expression networks were constructed using the blockwiseModules function in WGCNA.

The similarity matrix was then transformed into adjacency and topological overlap matrices (TOMs), with the latter serving as the basis for gene clustering through an average linkage hierarchical clustering approach. A hybrid dynamic tree-cutting algorithm was employed, specifying a minimum module size of 30 genes. Following module identification, module eigengenes (MEs) representing the first principal component of each module were calculated. Modules demonstrating high similarity were then merged based on a merge cut height of 0.25 and a deep split parameter of 2.

The expression profile of each module was summarized by its eigengene (ME), and Pearson correlation coefficients were computed to assess the associations between MEs and clinical trait vectors. Modules demonstrating stronger correlations were considered to have greater biological significance. Finally, ICD-associated hub modules were identified based on stringent criteria of module membership (MM > 0.7) and gene significance (GS > 0.6), highlighting the key co-expression networks linked to ICD-related hub genes.

#### Functional enrichment analyses of ICD-associated hub genes

The GO and KEGG enrichment analyses were performed using the clusterProfiler package in R. Multiple testing correction was applied using the Benjamini-Hochberg (BH) method, and pathways with a BH-adjusted *p*-value < 0.05 were considered statistically significant.

### ICD-related hub gene signature development

Univariate Cox proportional hazards regression analyses of ICD-associated hub genes were conducted using the coxph function from the survival package in R, applying a significance threshold of *p* < 0.05. To enhance the predictive robustness and minimize model overfitting, LASSO regression was subsequently performed using the glmnet package in R, reducing the number of coefficient variables and refining the prognostic model. Signature scores for each patient were determined according to the levels of gene expression and their regression coefficients with the formula:



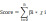



βi: gene weighting coefficient, χi: gene expression

### Examination of the correlations between signature scores and patient characteristics

#### Assessment of the prognostic utility of signature scores

Following LASSO regression analysis, patients were stratified into high- and low-score groups according to the median signature score. The prognostic performance of the constructed gene signature was subsequently evaluated using the survival, survminer, and pROC packages in R through Kaplan-Meier (KM) survival analysis and receiver operating characteristic (ROC) curve assessment.

#### Signature score distributions in relation to clinical characteristics

The distribution of signature scores in relation to various clinical characteristics within the training cohort was visualized using the ggplot2 package in R. Differences between groups were assessed using the Wilcoxon rank-sum test for two-group comparisons and the Kruskal-Wallis test for multiple-group analyses.

#### Univariate and multivariate Cox analyses for the training cohort

Clinical data from patients in the training cohort were subjected to univariate and multivariate Cox proportional hazards analyses using the coxph function from the survival package in R. A forest plot was generated to visualize the results, applying a significance threshold of *p* < 0.05. The independent prognostic value of the signature score was further evaluated based on its significance in both univariate and multivariate models.

### Characterization of signature score-related molecular features and drug responses

#### Assessment of signature score-related molecular features and pathway changes

The SNV and CNV data from the training cohort were analyzed after stratifying samples into high- and low-score groups based on whether their signature scores were above or below the median value. Hallmark gene sets retrieved from the MsigDB were used for pathway analysis, with TCGA gene expression data serving as input. Moreover, the ssGSEA scores for each pathway were computed using the GSVA package, and heatmaps illustrating the enrichment patterns of Hallmark pathways were generated with the pheatmap package in R.

#### Immune cell infiltration analyses

Gene sets representing characteristic expression profiles of distinct infiltrating immune cell types were obtained from a previously published study 14. In total, 28 immune cell types were analyzed, including regulatory T cells, natural killer T cells, activated dendritic cells, macrophages, and activated CD8⁺ T cells. The infiltration of immune and stromal cells within tumor tissues was inferred from gene expression data using the Pearson correlation method. Stromal and immune scores were calculated using the ESTIMATE package in R, and their combined value was defined as the ESTIMATE score. Immune cell infiltration levels were further quantified using the ssGSEA, CIBERSORT, and xCell algorithms. Differences in immune infiltration between high- and low-signature score groups were visualized and compared using the ggplot2 package in R, with the Wilcoxon rank-sum test employed for statistical evaluation.

#### Chemotherapy response analyses

The oncoPredict package in R was used to retrieve gene expression profiles and corresponding drug response data for cell lines from the GDSC2 database. Spearman rank correlation analyses were then performed to evaluate the relationships between signature scores (high- and low-score groups) and the log₁₀(IC₅₀) values of various drugs across different cell lines.

#### Analyses of immunotherapy responses

Due to the lack of available clinical datasets documenting immunotherapy responses in PAAD patients, the IMvigor210 cohort of advanced urothelial carcinoma was used as a surrogate for immunotherapy analysis. The previously established gene signature was applied to calculate a signature score for each sample, and KM survival curves were generated to compare survival outcomes between the high- and low-score groups. Furthermore, the distributions of responders (R) and non-responders (NoR) across these groups were analyzed to assess the potential predictive value of the signature for immunotherapy responsiveness.

### Experimental validation

AJM1 expression levels were validated by quantitative PCR (qPCR) in an internal cohort of patients who underwent surgical resection at the Department of Hepatobiliary & Pancreatic Surgery, Zhongnan Hospital of Wuhan University, between 2020 and 2023. None of the patients received neoadjuvant chemotherapy before surgery, and written informed consent was obtained from all participants. The study was conducted in accordance with the Declaration of Helsinki and was approved by the Medical Ethics Committee of Zhongnan Hospital of Wuhan University (approval number: 2025005K). Clinical characteristics of the enrolled patients are summarized in **Table S4**. Further validation of AJM1 protein expression was performed using immunohistochemistry (IHC) data from the Human Protein Atlas (HPA) database.

To further explore the functional role of AJM1 in pancreatic cancer cells, AsPC-1 PAAD cells were transfected with siAJM1, followed by Cell Counting Kit-8 (CCK-8), 5-ethynyl-2′-deoxyuridine (EdU) incorporation, and colony formation assays, conducted according to established protocols 15-18. All primer and siRNA sequences used in this study are provided in **Table S5**.

## Results

### Characterization of the ICD gene landscape

A total of 181 tissue samples, including 177 tumor and 4 normal tissues, from the TCGA-PAAD cohort were analyzed to characterize the landscape of ICD-related genes in pancreatic adenocarcinoma. The expression profiles of 34 ICD genes were compared between groups using Wilcoxon rank-sum tests (**Fig. S1A**). Among these, 10 genes (*CD4*, *IFNGR1*, *P2RX7*, *TLR4*, *ENTPD1*, *LY96*, *NLRP3*, *IL17RA*, *PRF1*, and *TNF*) were significantly downregulated in tumor samples, whereas *PDIA3* was significantly upregulated.

Further subgroup analyses revealed significant clinical correlations. Expression levels of *IFNGR1*, *CASP8*, *CASP1*, and *MYD88* were significantly higher in stage II/III/IV tumors compared to stage I (**Fig. S1B**). Similarly, *PIK3CA* and *NT5E* expression levels were elevated in patients younger than 60 years compared with those aged 60 years or older (**Fig. S1C**). Female patients showed significantly increased expression of *IFNGR1*, *FOXP3*, *IL1B*, *IL6*, *NLRP3*, and *TNF* relative to males (**Fig. S1D**). Moreover, grade 3/4 tumors showed higher expression of *CASP8* and *NT5E* than grade 1/2 tumors (**Fig. S1E**). *PIK3CA* and *NT5E* expression levels were also significantly elevated in tumor-positive compared with tumor-free samples (**Fig. S1F**). Further, eight genes (*CD4*, *IFNGR1*, *CASP8*, *PIK3CA*, *IL10*, *LY96*, *MYD88*, and *CXCR3*) showed pronounced expression differences across tissue types (**Fig. S1G**). *IFNGR1* demonstrated significant variability across multiple parameters, including tissue type, tumor stage, sex, and histological subtype, highlighting its potential clinical relevance in PAAD.

The SNV landscape of ICD genes was also examined (**Fig. S2A**), revealing an overall low mutation frequency across the training cohort. Among all ICD genes, *PIK3CA* displayed the highest mutation incidence, and all detected variants were missense mutations. The chromosomal localization of the ICD genes is illustrated in **Fig. S2B**. Furthermore, the CNV profiles of these genes were analyzed (**Fig. S2C**), indicating a relatively higher frequency of CNV alterations in *IFNA1*, predominantly characterized by copy number deletions.

### Identification and functional enrichment analyses of ICD-associated hub genes in PAAD

**Figure 1:**
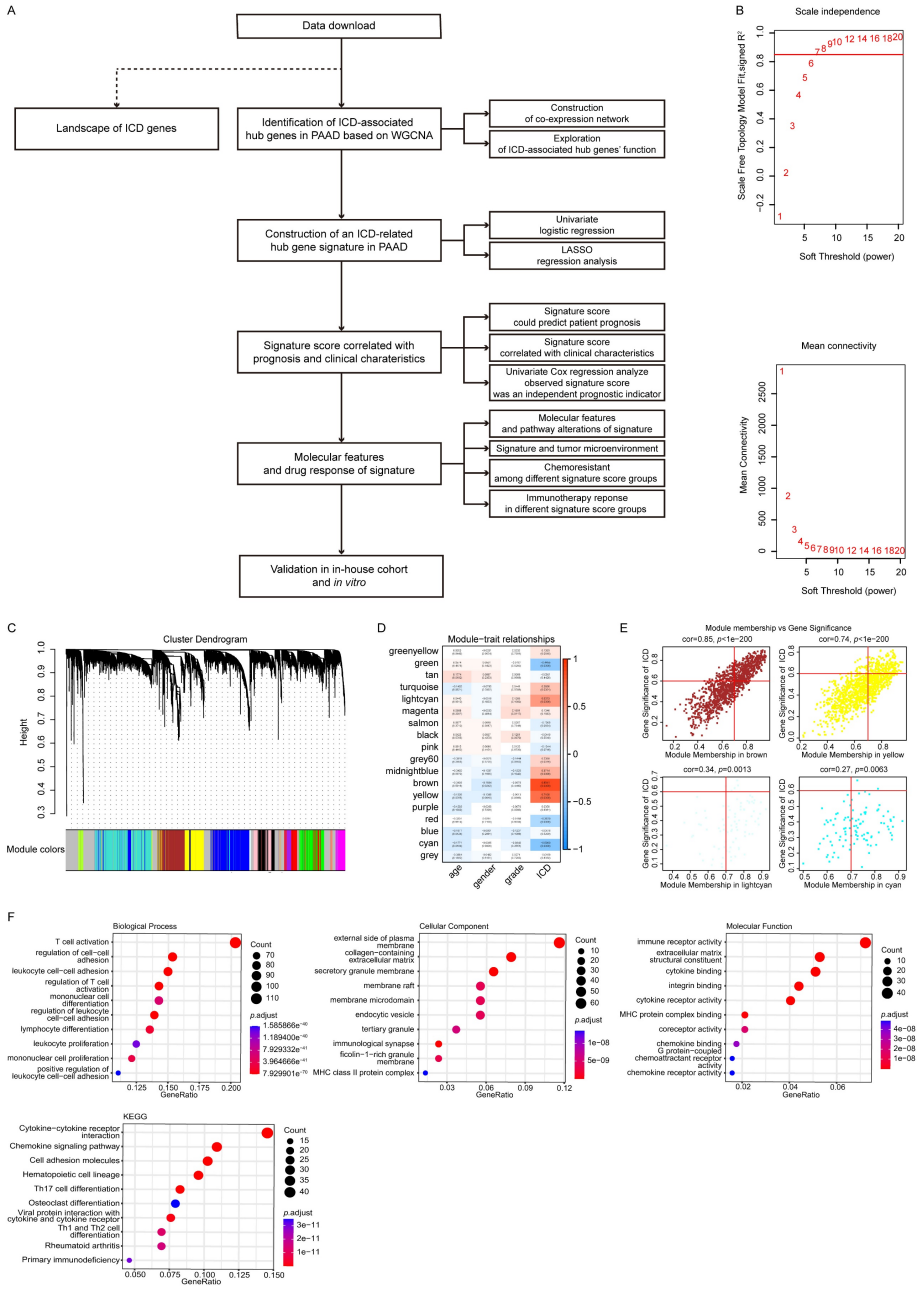
** Identification and functional enrichment analyses of ICD-associated hub genes in PAAD. (A)** Study flow chart. The training dataset was obtained from TCGA-PAAD, and the validation dataset was GSE57495 from GEO. **(B)** Network topology analyses for different soft-thresholding powers. **(C)** A gene dendrogram and module colors. **(D)** Module-trait correlations. **(E)** Correlations between MM and GS for the brown, yellow, cyan, and light cyan modules. **(F)** GO and KEGG pathway enrichment analyses.

To identify ICD-associated hub genes, data from the training cohort were first analyzed using WGCNA (**Fig. 1A**). The ssGSEA algorithm was applied to determine an appropriate soft threshold power (β). ICD levels in individual samples were quantified based on the resulting enrichment scores (**Fig. 1B-C**). Pearson correlation analysis was then conducted to assess the relationships between MEs and sample trait feature vectors. Among the constructed modules, the brown, yellow, cyan, and light cyan modules showed the strongest positive correlations with ICD levels and were therefore selected for subsequent analyses. Applying stringent screening criteria ([MM] > 0.7 and [GS] > 0.6), a total of 606 ICD-associated hub genes were identified for downstream functional characterization (**Fig. 1D-E**).

Functional enrichment analyses were then performed on these 606 genes using GO and KEGG databases (**Fig. 1F**). The GO analysis revealed significant enrichment in biological processes related to T cell activation and regulation of cell-cell adhesion. At the same time, cellular component enrichment was observed for the external side of the plasma membrane and the collagen-containing extracellular matrix. In terms of molecular functions, these genes were predominantly associated with immune receptor activity and structural constituents of the extracellular matrix. Consistent with these findings, KEGG pathway analysis indicated that the cytokine-cytokine receptor interaction and chemokine signaling pathways were among the most significantly enriched pathways, suggesting that these hub genes play crucial roles in immune modulation and tumor-microenvironment interactions.

### Establishment of an ICD-related hub gene signature in PAAD

**Figure 2:**
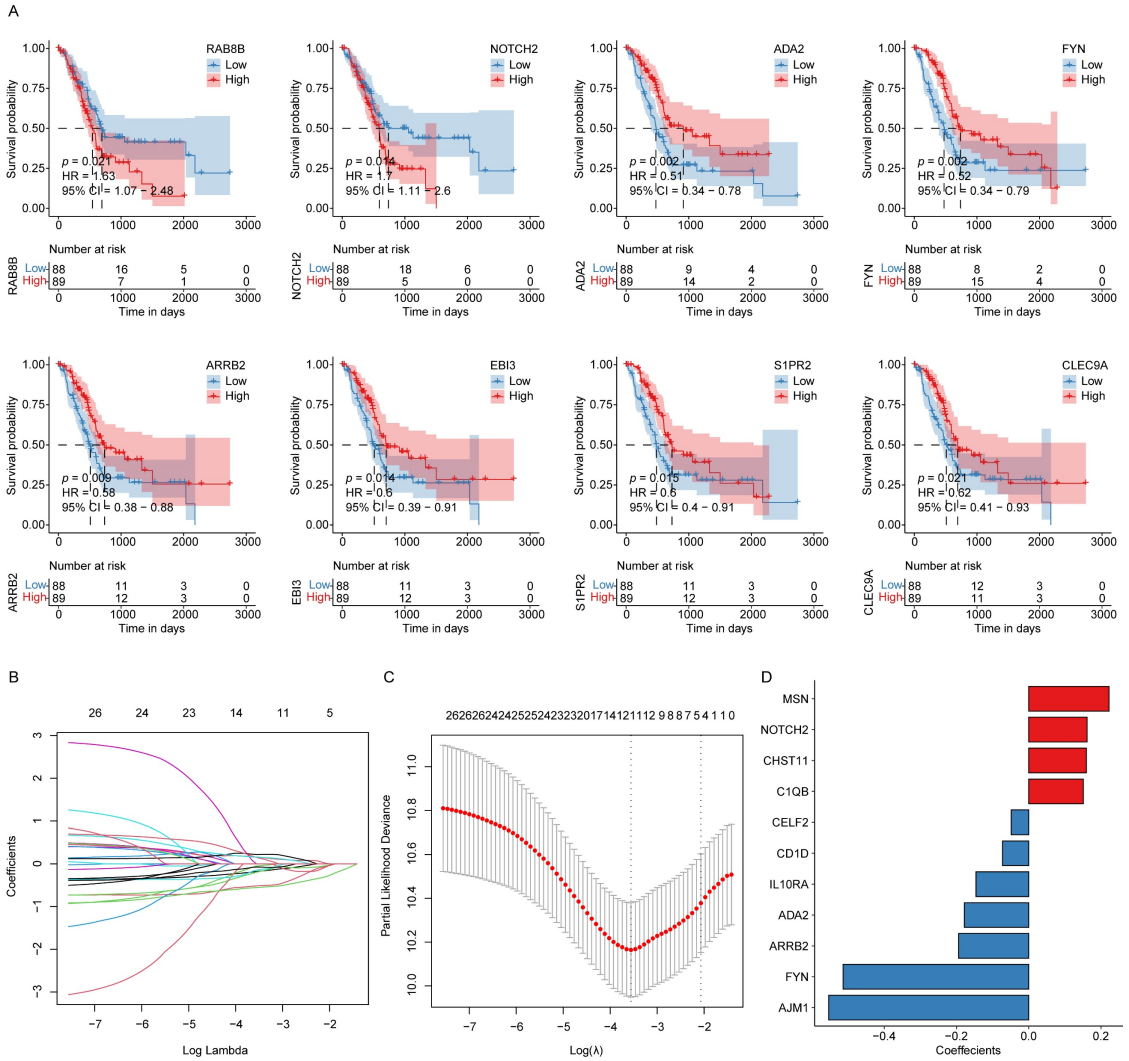
** ICD-related hub gene signature construction in PAAD. (A)** KM survival curves for those genes with significant differences between the high- and low-ICD groups. **(B)** LASSO regression analysis trajectories for the 26 independent genes, with the log of the lambda value for these independent variables along the x-axis, while the y-axis shows the coefficients for these independent variables. **(C)** Confidence intervals corresponding to each lambda. **(D)** LASSO coefficient profiles for the 11 key prognostic genes identified through this approach.

Using the 606 ICD-associated hub genes identified from the preceding WGCNA analysis, a univariate Cox regression was conducted based on data from 176 patient samples. Applying appropriate statistical thresholds, 26 genes with potential prognostic significance were identified (**Table S6**). For each gene, the median expression value served as a cutoff to stratify samples into high- and low-expression groups. The KM survival analyses were then performed to evaluate overall survival (OS) differences between these groups. The results demonstrated that low expression levels of *RAB8B* and *NOTCH2* were significantly correlated with prolonged OS, whereas higher expression levels of *ADA2*, *FYN*, *ARRB2*, *EBI3*, *S1PR2*, and *CLEC9A* were indicative of better patient prognosis (**Fig. 2A**).

Afterward, these 26 candidate genes were subjected to LASSO regression analysis, which further refined the list to 11 key prognostic genes associated with PAAD outcomes. These genes included *MSN*, *NOTCH2*, *CHST11*, *C1QB*, *CELF2*, *CD1D*, *IL10RA*, *ADA2*, *ARRB2*, *FYN*, and *AJM1*. As illustrated in **Fig. 2B-D**, the LASSO regression coefficients corresponding to these 11 genes were used to weight their expression levels, constructing a robust ICD-related prognostic gene signature for stratifying PAAD patient samples.

### Signature scores are related to PAAD patient prognosis

**Figure 3:**
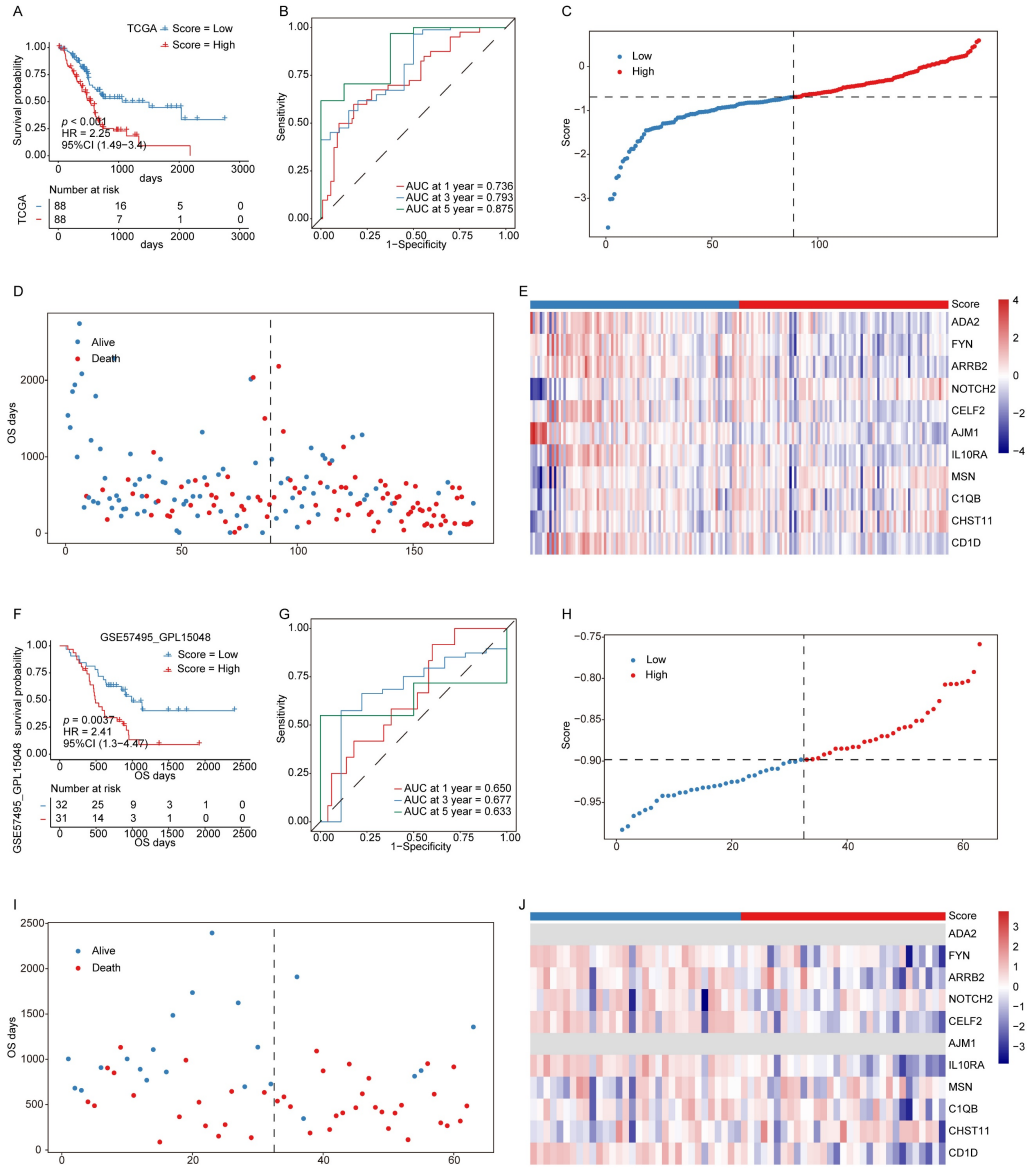
** Signature scores are associated with PAAD patient prognosis. (A, B)** KM survival curves (A) and ROC curves (B) for the training cohort. **(C)** Signature score distributions for the training cohort. **(D)** Survival time distributions in the training cohort. **(F, G)** KM survival curves (F) and ROC curves (G) for the validation cohort. **(H)** Signature score distributions for the validation cohort. **(I)** Survival time distributions in the validation cohort. **(J)** Expression levels of 11 key prognostic factors in the validation cohort.

The constructed ICD-related hub gene signature was applied to calculate a signature score for each patient sample. The KM and ROC curve analyses in both the training and validation cohorts demonstrated that lower signature scores were significantly associated with a more favorable prognosis (**Fig. 3A-B, F-G**). As shown in **Figs. 3C** and **3H**, the distribution of signature scores across both cohorts revealed a continuous pattern, with no evident outliers or extreme values. Similarly, in the survival analyses, patients classified into the low-score group showed prolonged OS compared with those in the high-score group (**Fig. 3D, I**).

Expression levels of the 11 genes constituting the prognostic model were also evaluated in both cohorts (**Fig. 3E, J**), revealing concordant expression patterns between the two datasets, except for a few genes that were undetectable in the validation cohort. These findings indicate that the established signature score serves as a robust and reliable prognostic biomarker for PAAD.

### Signature score values serve as independent predictors of PAAD patient prognosis

**Figure 4:**
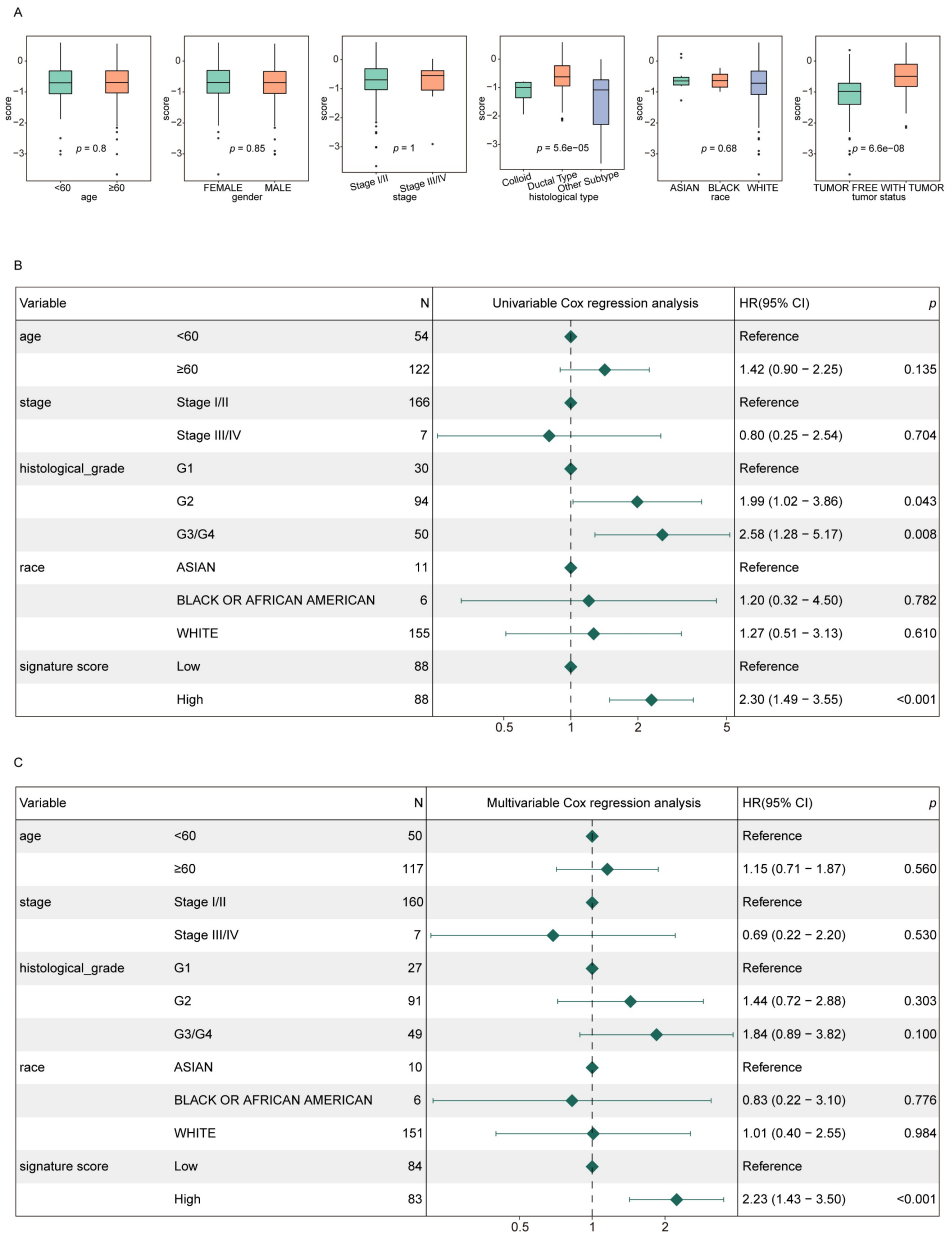
** Signature scores are independently associated with PAAD patient prognosis. (A)** Signature score distributions for different clinical features. **(B, C)** Forest plots for univariate (B) and multivariate (C) regression analyses in the training cohort.

To evaluate the independent prognostic value of the signature score in PAAD patients, score distributions were analyzed across various clinical subgroups, excluding those with fewer than two samples (**Fig. 4A**). The analysis revealed that signature scores varied significantly with respect to tumor status and histological subtype. Patients classified as WITH TUMOR showed significantly higher signature scores compared to those categorized as TUMOR FREE. Similarly, ductal-type tumors displayed higher signature score values than other histological variants.

Furthermore, both univariate and multivariate Cox regression analyses demonstrated that the signature score was independently correlated with the prognosis of PAAD patients (*p* < 0.05), reinforcing its potential as an independent prognostic indicator (**Fig. 4B-C**).

### Evaluation of signature score-related molecular features and changes in pathway activities

**Figure 5:**
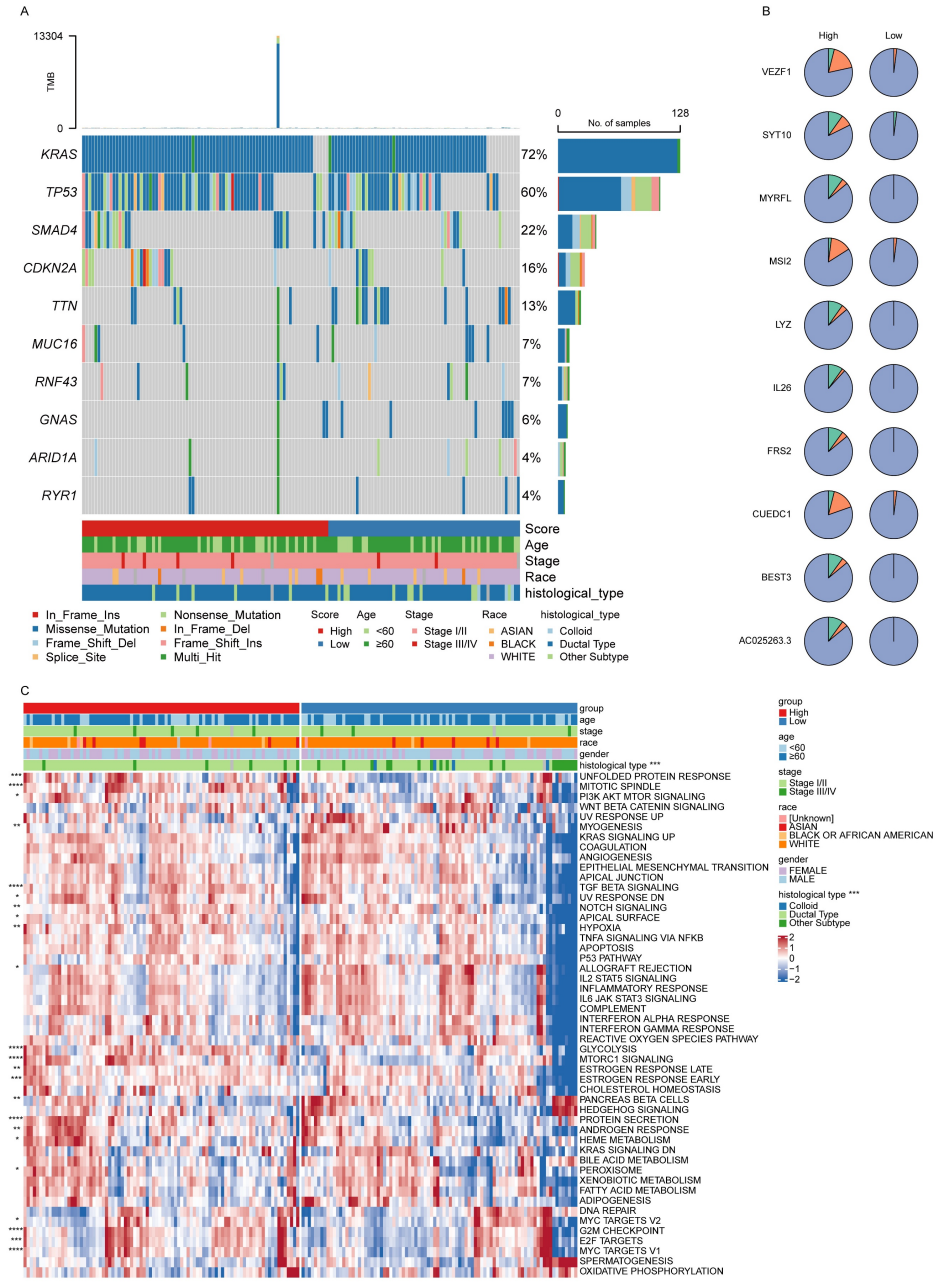
** Signature score-related molecular features and pathway enrichment. (A, B)** The top 10 SNVs (A) and CNVs (B) in the low- and high-score groups. **(C)** Differences in pathway enrichment levels in the low- and high-score groups.

The top 10 most prevalent SNVs and CNVs were subsequently compared between the high- and low-score patient groups. Among these, *KRAS* and *TP53* emerged as the most frequently mutated genes, with *KRAS* predominantly demonstrating missense mutations (**Fig. 5A**). Furthermore, the overall frequency of CNVs was significantly higher in the high-score group than in the low-score group (**Fig. 5B**).

To further explore biological pathway differences between these two groups, a ssGSEA was performed. This analysis revealed a significant variation in pathway activity (**Fig. 5C**), demonstrating enhanced enrichment of the mitotic spindle and TGF-β signaling pathways in the high-score group, indicating their potential involvement in PAAD progression.

### PAAD patient signature scores are associated with tumor microenvironment composition

**Figure 6:**
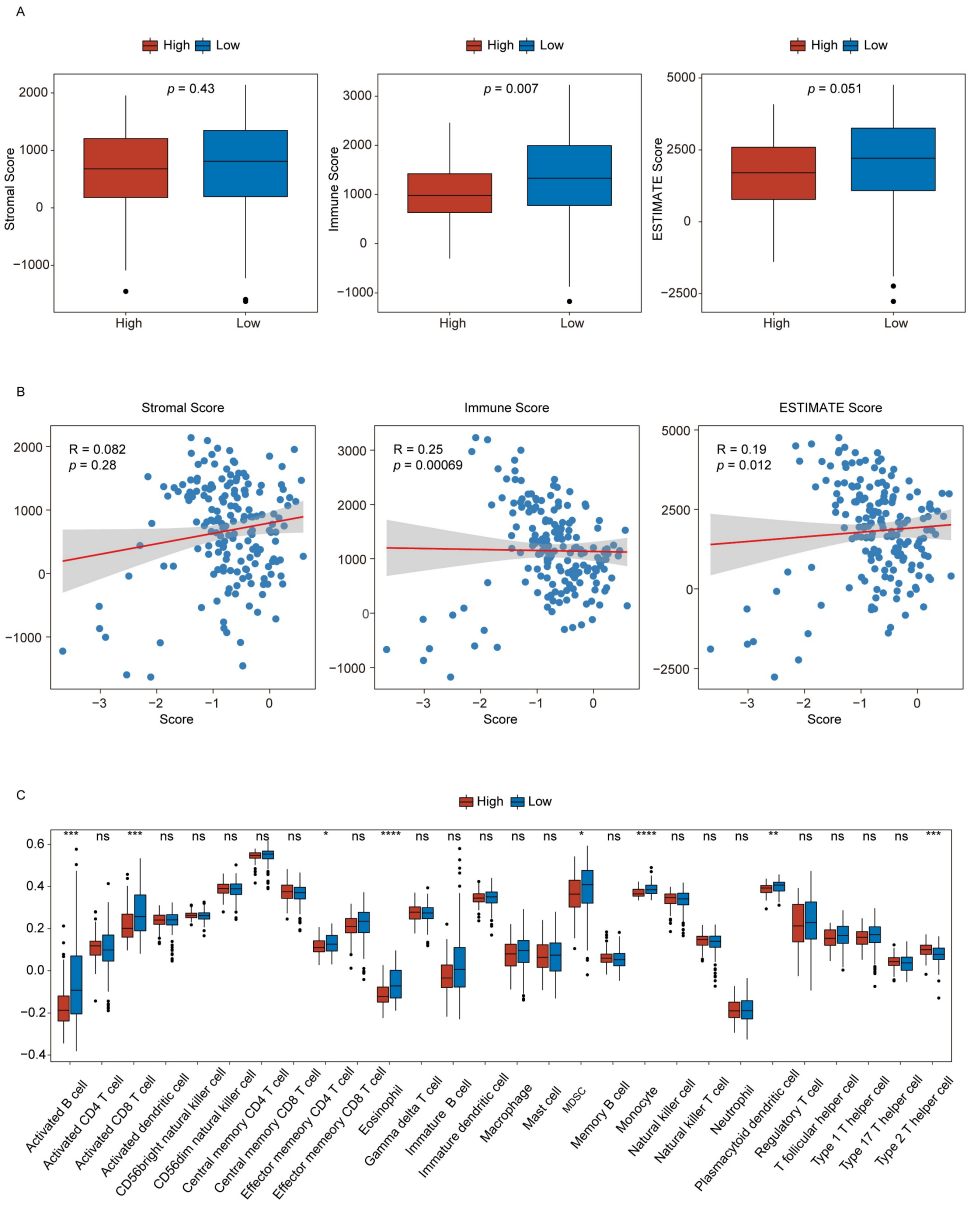
** Signature scores offer utility for the evaluation of the tumor microenvironment. (A)** Stromal, immune, and ESTIMATE scores in the low- and high-score groups. **(B)** Correlations between signature scores and stromal, immune, and ESTIMATE scores. **(C)** Immune cell infiltration levels in the high- and low-score groups. n.s., not significant, *p ≤ 0.05, **p ≤ 0.01, ***p ≤ 0.001 and ****p ≤ 0.0001.

To evaluate the relationship between the signature score and the TME composition in PAAD patients, stromal, immune, and ESTIMATE scores were computed using the R ESTIMATE package. Their distributions were compared between the low- and high-score groups (**Fig. 6A**). The analysis revealed that immune scores were significantly lower in the high-score group than in the low-score group, indicating a relative suppression of immune activity. Furthermore, correlation analyses demonstrated that signature scores were positively associated with both stromal and ESTIMATE scores (**Fig. 6B**).

The infiltration profiles of immune cell populations were next examined via ssGSEA to compare differences between the two groups (**Fig. 6C**). The results showed that seven immune cell types, including activated B cells and activated CD8⁺ T cells, showed reduced infiltration in the high-score group. In comparison, Th2 cell infiltration was elevated in these same samples. These findings suggest that higher signature scores may reflect a more immunosuppressive TME phenotype in PAAD.

### Signature scores are associated with potential chemoresistance and immunotherapy responses

**Figure 7:**
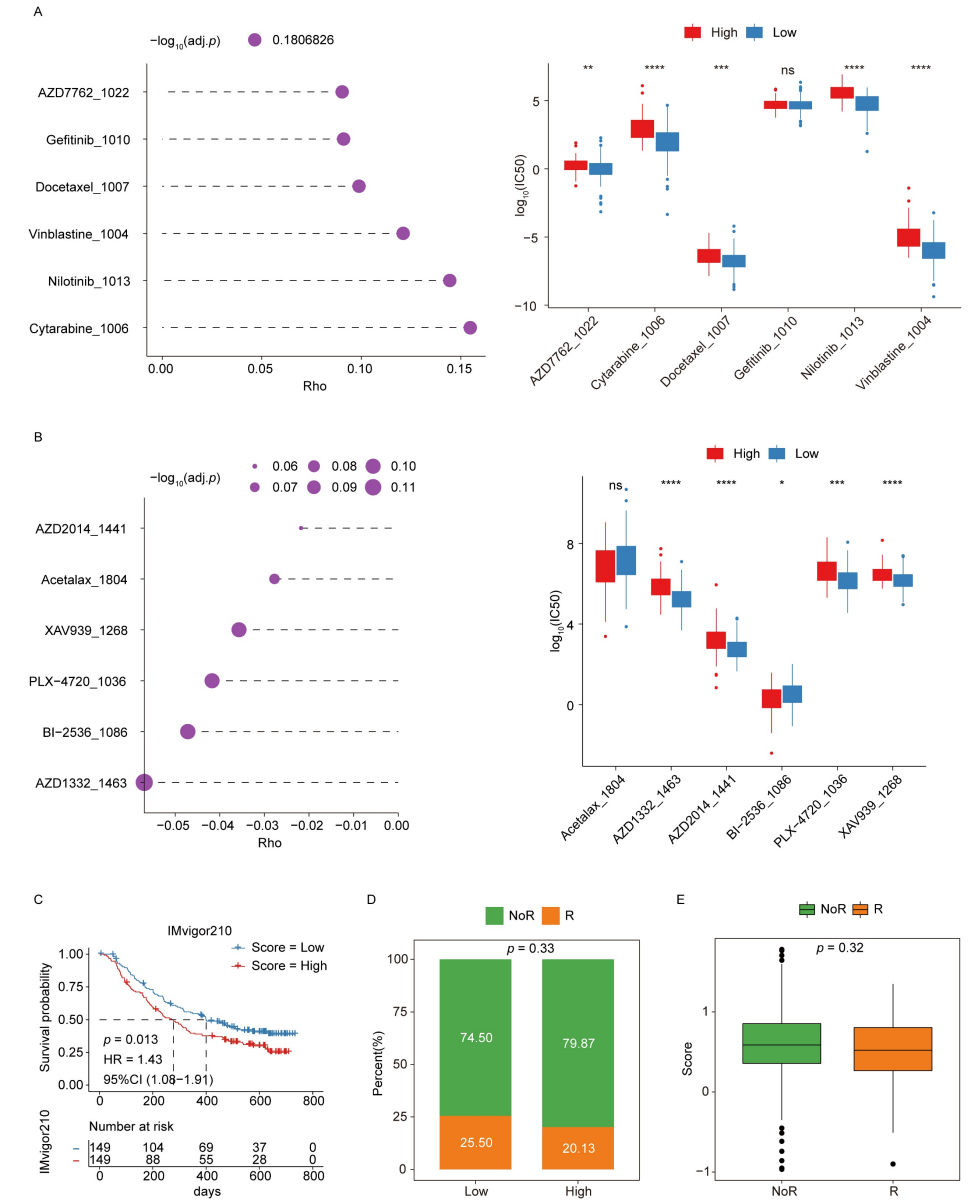
** Signature scores are associated with chemoresistance and immunotherapy responses. (A-B)** Analyses of chemoresistance in the low- and high-score groups. The top 6 drugs with the strongest positive (**A**) or negative (**B**) correlations with signature scores are presented, along with their corresponding *p-*values. **(C)** KM curves for the immunotherapeutic advanced urothelial cancer cohort (IMvigor210). **(D)** Responder and non-responder distributions in the low- and high-score groups. **(E)** Differences in responses between the low- and high-score groups.

To explore the association between chemoresistance and the signature score, analyses were performed using cell line expression and drug response data from the GDSC2 database. Spearman correlation coefficients were calculated to evaluate the relationships between log_10_(IC_50_) values for individual drugs and signature score values, which were derived from the corresponding gene expression profiles of each cell line. The analysis revealed significant differences in correlation coefficients between the low- and high-scoring groups, suggesting distinct patterns of drug sensitivity (**Fig. 7A-B**).

Afterward, the potential of the ICD-related hub genes to predict immunotherapy response was assessed using data from the IMvigor210 cohort. Kaplan-Meier survival analyses demonstrated that patients in the low-score group showed a more favorable prognosis following immunotherapy (**Fig. 7C**). Moreover, comparisons of responder (R) and non-responder (NoR) distributions indicated a higher proportion of responders in the low-score group, supporting the predictive value of the signature score for immunotherapeutic efficacy (**Fig. 7D-E**).

### Validation of AJM1 as a novel prognostic gene in PAAD

**Figure 8:**
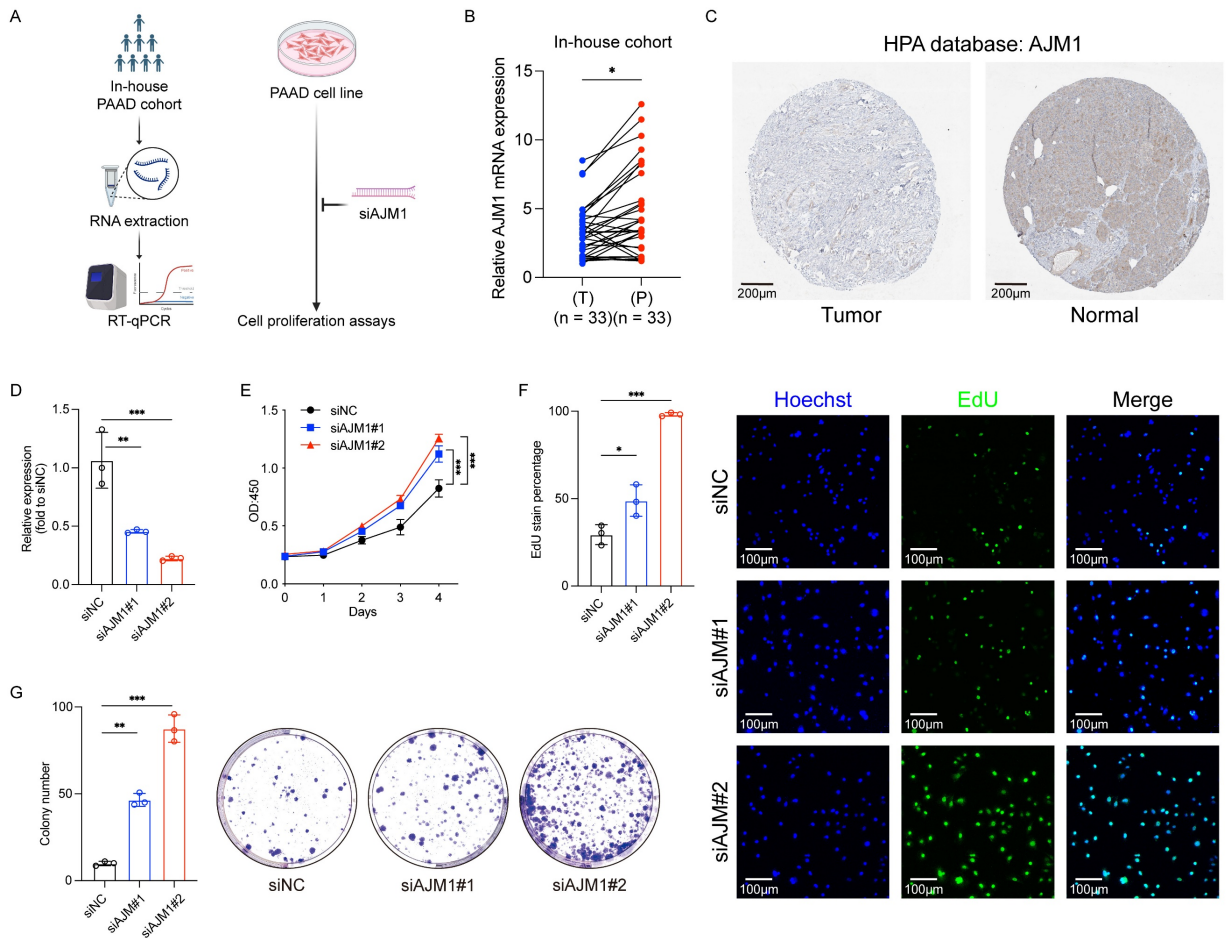
** Validation of AJM1 as a novel protective gene in PAAD. (A)** Experimental workflow for the internal patient cohort and *in vitro* analyses. **(B)** qPCR-based analysis of the expression of AJM1 in the internal cohort. T: Tumor tissue, P: Paracancerous tissue. **(C)** AJM1 immunohistochemical staining results in the HPA database. **(D)** qPCR-based validation of the siRNA transfection efficiency levels in AsPC-1 cells. **(E)** CCK-8 assay. **(F)** EdU assay and corresponding analyses of the frequency of EdU-positive cells. **(G)** Colony formation assay and corresponding quantification. n.s., not significant, *p ≤ 0.05, **p ≤ 0.01, ***p ≤ 0.001 and ****p ≤ 0.0001.

Based on the successful development of the ICD-related hub gene signature as a prognostic and drug response predictor in PAAD, further validation analyses were conducted focusing on the model genes. Using 33 pairs of PAAD tumor and adjacent non-tumor tissue samples (**Fig. 8A**), qPCR analyses confirmed that *AJM1* expression was significantly higher in paracancerous tissues compared with tumor tissues (**Fig. 8B**). These findings were further supported by IHC results demonstrating a similar expression trend (**Fig. 8C**).

In *in vitro* functional assays, *AJM1* knockdown was achieved via siRNA transfection in AsPC-1 PAAD cells (**Fig. 8D**). Results from CCK-8, EdU incorporation, and colony formation assays consistently showed that silencing *AJM1* significantly increased PAAD cell proliferation (**Fig. 8E-G**). These findings confirm the functional significance of *AJM1* in the progression of PAAD and underscore its potential as a prognostic biomarker and therapeutic target in this malignancy.

## Discussion

The PAAD remains one of the most lethal malignancies, mainly due to the absence of reliable early diagnostic biomarkers and its frequent detection at advanced stages, where tumors show significant resistance to conventional therapies. A defining characteristic of PAAD is its remarkable ability to evade immune surveillance, which severely limits the efficacy of chemotherapeutic and radiotherapeutic approaches 19-21. The lack of robust prognostic biomarkers further constrains opportunities for personalized therapeutic strategies, underscoring the urgent need for novel molecular indicators that can guide clinical decision-making.

Emerging evidence highlights the tumor immune microenvironment (TIME) as a key driver of PAAD progression, orchestrated through complex interactions between tumor and immune cells 22-24. Within this context, ICD, a regulated form of cell death that provokes both innate and adaptive immune responses, has garnered increasing attention as a potential therapeutic avenue 25,26. ICD promotes the release of damage-associated molecular patterns (DAMPs) and tumor-associated antigens, increasing the activation of immune cells and tumor-specific cytotoxicity 27. Although preclinical studies have demonstrated that ICD can foster sustained anti-tumor immunity and increase responsiveness to immunotherapy, clinical validation in PAAD remains limited, emphasizing the need for further translational research to clarify its prognostic and therapeutic potential.

AJM1 is a critical tight junction (TJ) protein responsible for maintaining epithelial integrity and regulating cellular signaling pathways within the intestinal barrier 28. It interacts with structural proteins such as VAB-9 and DLG-1, contributing to the maintenance of junctional stability and epithelial polarity 29. Under proteotoxic stress conditions, disruptions in AJM1 localization mediated by mTOR sequestration and excessive autophagy activation can compromise epithelial integrity, underscoring its sensitivity to cellular stress 30. Recent evidence has linked AJM1 expression to intestinal disease models and toxicological stress responses. For instance, litchi polysaccharides have been shown to protect intestinal barrier function, partly by modulating AJM1 expression, in *C. elegans* and murine models 31. Similarly, exposure to mycotoxins such as tenuazonic acid and patulin disrupted AJM1 expression in *C. elegans*, suggesting that it may serve as a mediator of intestinal stress responses 32. These studies indicate that AJM1 may serve as a biomarker for digestive tract pathophysiology. Moreover, tight junction proteins, by preserving epithelial polarity, may act to suppress uncontrolled proliferation and migration associated with the EMT, a hallmark of tumor progression. However, the specific functional role of AJM1 in PAAD has remained poorly defined.

In this study, the relationship between ICD and PAAD prognosis was systematically examined through the analysis of ICD-associated hub genes identified using ssGSEA and WGCNA. The constructed ICD-related hub gene signature effectively stratified PAAD patients into low- and high-score groups with distinct prognostic outcomes. This signature demonstrated independent prognostic value in both univariate and multivariate Cox regression analyses, while also revealing significant differences in tumor mutational burden (SNVs and CNVs) and immune cell infiltration patterns between the two groups. Immune scores and immune infiltration levels were significantly higher in the low-score group, suggesting a more immunologically active tumor microenvironment, which may contribute to improved clinical outcomes and increased therapeutic responsiveness.

The present study builds upon and extends a recent report 33 that employed consensus clustering to categorize PAAD samples according to ICD-related gene expression while characterizing the immune landscape using data from 502 HNSCC samples. Although that approach broadened the general applicability of the ICD gene signature across multiple cancer types, it may not have fully captured the immunosuppressive characteristics of the PAAD tumor microenvironment, which significantly contrasts with the immune-active (hot) phenotype typical of HNSCC. In comparison, our use of WGCNA facilitated the identification of PAAD-specific ICD-associated hub gene modules, improving disease specificity. By exclusively analyzing PAAD-derived datasets, the resulting gene signature was optimized for evaluating the tumor immune microenvironment in this cancer type. These methodological refinements enhance the precision and translational relevance of our findings, offering a valuable complement to the previous study.

The drug-sensitivity analyses further underscored the clinical relevance of the ICD-related hub gene signature established in this study. By calculating Spearman correlation coefficients between the signature scores and IC_50_ values from the GDSC2 database, distinct patterns of drug sensitivity were identified, offering potential strategies to optimize therapeutic regimens for patients in the low- and high-score groups.

The protective function of *AJM1* is further supported by its potential role in modulating the tumor microenvironment. In this study, qPCR analyses revealed significantly higher *AJM1* expression in paracancerous tissues compared to tumor tissues, suggesting that its downregulation may contribute to tumor initiation and progression. Similarly, *in vitro* assays, including CCK-8, EdU incorporation, and colony formation, demonstrated that silencing *AJM1* significantly increased the proliferation of PAAD cells. These findings suggest that *AJM1* functions as a tumor suppressor, limiting the proliferative capacity of PAAD cells.

In conclusion, this study establishes an ICD-related hub gene signature, constructed using machine learning, that effectively predicts prognostic outcomes in patients with PAAD. This signature offers a promising framework for the personalized management and therapeutic stratification of this highly aggressive malignancy. Moreover, the observed link between *AJM1* downregulation and increased tumor cell proliferation underscores the potential importance of epithelial junction regulation as a therapeutic target in PAAD. However, further clinical validation will be necessary to confirm the predictive and translational value of the proposed signature in broader patient cohorts.

## Supplementary Material

Supplementary figures and tables.

## Abbreviations

PAAD: Pancreatic adenocarcinoma
ICD: Immunogenic cell death
ssGSEA: Single-sample gene set enrichment analysis
WGCNA: Weighted gene co-expression network analysis
MsigDB: Molecular Signatures Database
SNVs: Single-nucleotide variations
CNVs: Copy number variations
TOM: Topological overlap matrix
ME: Module eigengene
MM: Module membership
GS: Gene significance
GO: Gene Ontology
KEGG: Kyoto Encyclopedia of Genes and Genomes
BH: Benjamini-Hochberg
KM: Kaplan-Meier
ROC: Receiver operating characteristic
R: Responder
NoR: Non-Responder
IHC: Immunohistochemistry
HPA: Human Protein Atlas
CCK-8: Cell Counting Kit-8
EdU: 5-Ethynyl-2'-deoxyuridine
OS: Overall survival
T: Tumor
P: Paracancerous tissue
