# Supplementary material for: Machine Learning-Based WGCNA Approach for Developing an Immunogenic Cell Death-Related Hub Gene Signature and Identification of AJM1 as a Prognostic Biomarker in Pancreatic Adenocarcinoma
Source: Int J Med Sci. 2025 Oct 27;22(16):4493–508. doi: 10.7150/ijms.119960 (PMC12595326; doi:10.7150/ijms.119960)
Supplement: Supplementary file 1 — Supplementary figures and tables. [file ijmsv22p4493s1.pdf]

Supplementary Materials for

Machine Learning-Based WGCNA Approach for Developing an Immunogenic Cell Death-Related Hub Gene Signature and Identification of AJM1 as a Prognostic Biomarker in Pancreatic Adenocarcinoma

Tianyin Ma *et al.*

*Corresponding author. Email: wangqikun131@163.com (Q.W.);

dr.med.mingtian@whu.edu.cn (M.T.)

This file includes:

Figs. S1 to S2
Table S1 to S6

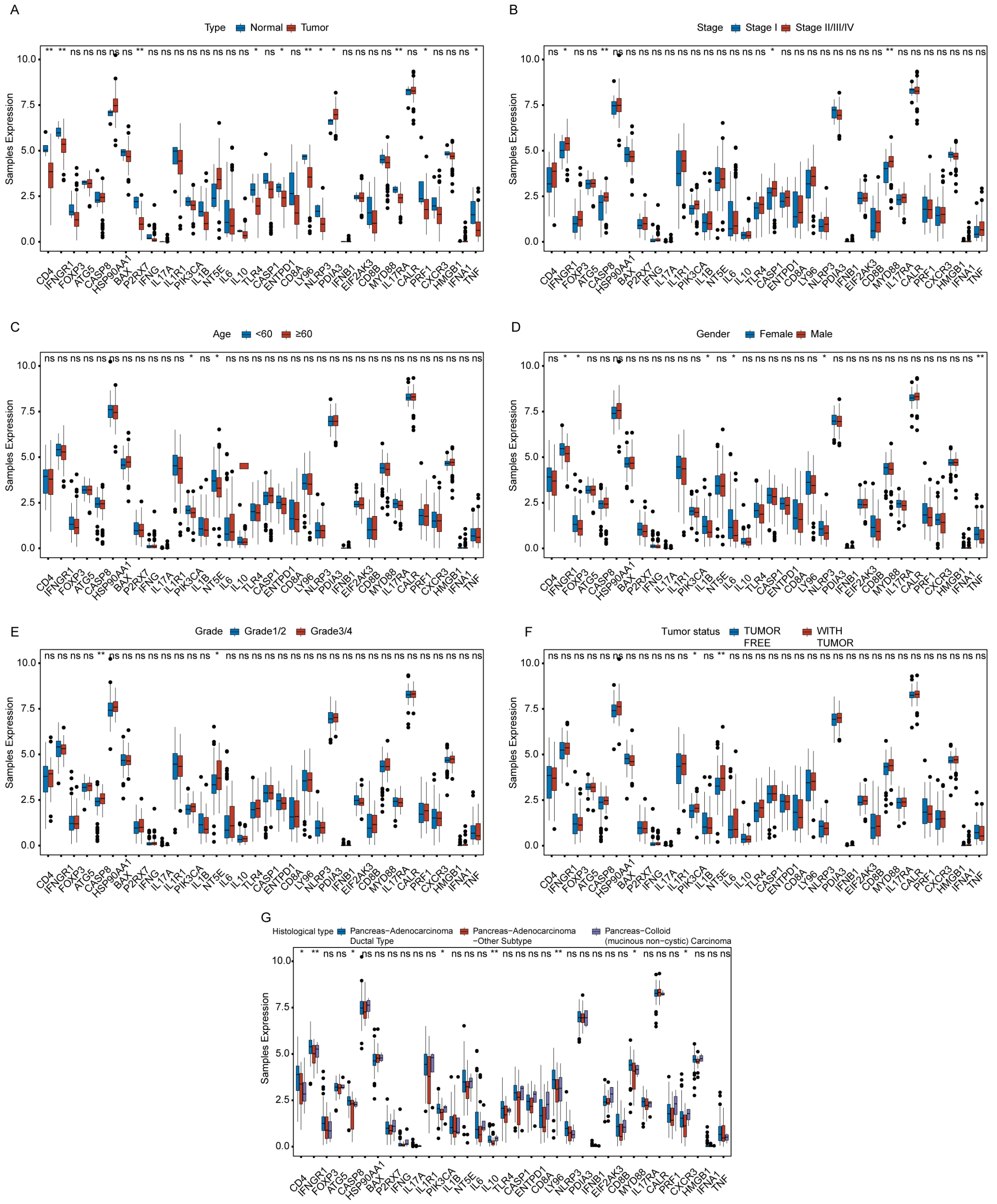

A
Type Normal Tumor
B
Stage Stage I Stage II/III/IV
C
Age <60 ≥60
D
Gender Female Male
E
Grade Grade1/2 Grade3/4
F
Tumor status TUMOR FREE WITH TUMOR
G
Histological type Pancreas−Adenocarcinoma Ductal Type Pancreas−Adenocarcinoma −Other Subtype Pancreas−Colloid (mucinous non−cystic) Carcinoma
Samples Expression
10.0
7.5
5.0
2.5
0.0
CD4 IFNGR1 FOXP3 ATG5 CASP8 HSP90AA1 BAX P2RX7 IFNG IL17A IL1R1 PIK3CA IL1B NT5E IL6 IL10 TLR4 CASP1 ENTPD1 CD8A LY96 NLRP3 PDIA3 IFNB1 EIF2AK3 CD8B MYD88 IL17RA CALR PRF1 CXCR3 HMGB1 IFNA1 TNF

Fig. S1. ICD gene expression profiles in different groups

(A) Differential ICD gene expression in the normal and tumor sample groups. **(B-G)** Differential expression of ICD genes as a function of stage (B), age (C), gender (D), grade (E), tumor status (F), and histological type (G). n.s., not significant, $^*p \leq 0.05$, $^{**}p \leq 0.01$, $^{***}p \leq 0.001$ and $^{****}p \leq 0.0001$.

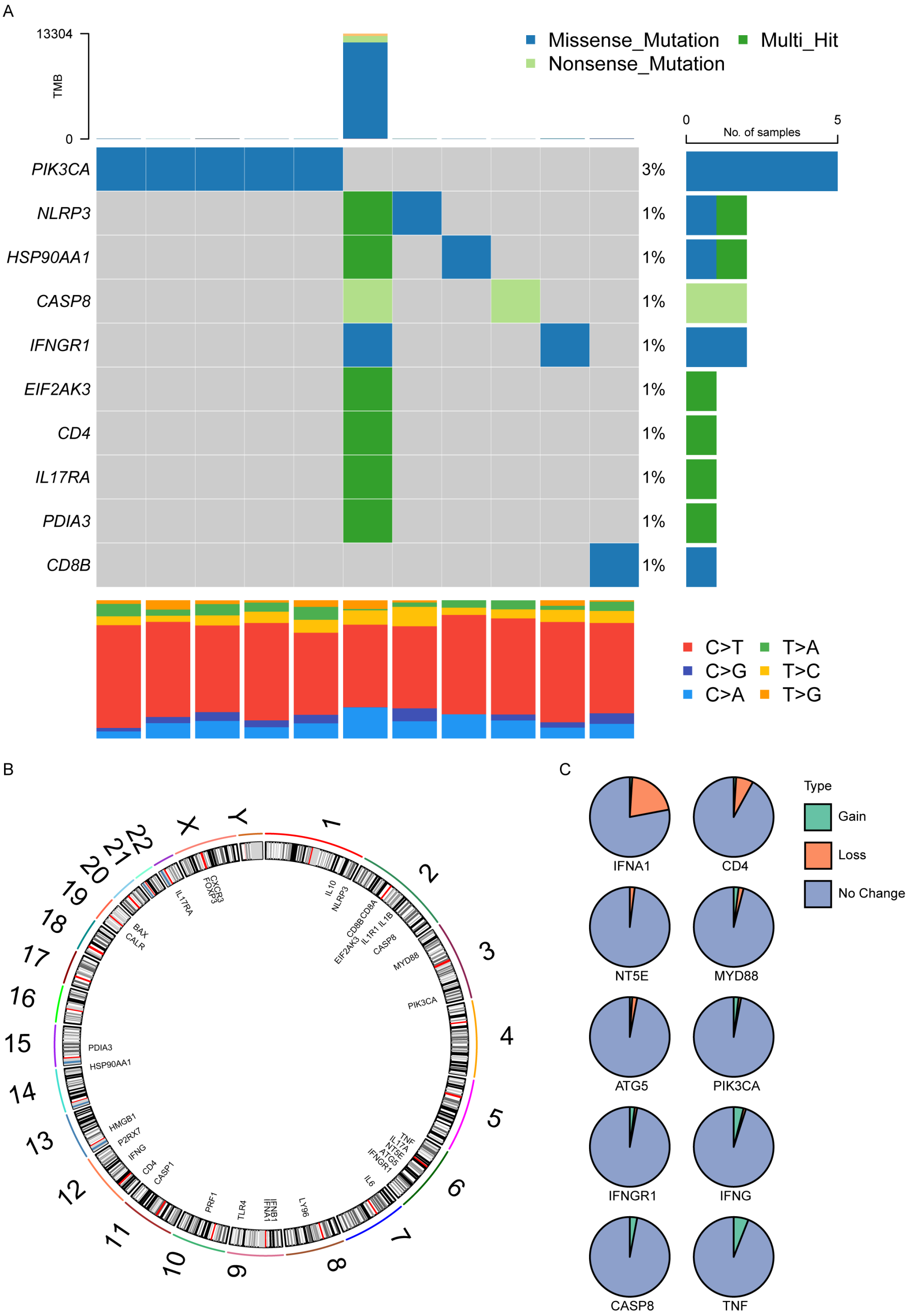
A
13304
TMB
0
Missense_Mutation
Multi_Hit
Nonsense_Mutation
0
5
No. of samples
PIK3CA
NLRP3
HSP90AA1
CASP8
IFNGR1
EIF2AK3
CD4
IL17RA
PDIA3
CD8B
3%
1%
1%
1%
1%
1%
1%
1%
1%
1%
C>T
T>A
C>G
T>C
C>A
T>G
B
1
2
3
4
5
6
7
8
9
10
11
12
13
14
15
16
17
18
19
20
21
22
X
Y
IL10
NLRP3
CD8B
CD8A
IL1B
EIF2AK3
IL1R1
CASP8
MYD88
PIK3CA
TNF
IL17A
NT5E
ATG5
IFNGR1
IL6
LY96
IFNB1
IFNA1
TLR4
PRF1
CASP1
CD4
IFNG
P2RX7
HMGB1
HSP90AA1
PDIA3
BAX
CALR
IL17RA
CXCR3
FOXP3
C
Type
Gain
Loss
No Change
IFNA1
CD4
NT5E
MYD88
ATG5
PIK3CA
IFNGR1
IFNG
CASP8
TNF

Fig. S2. ICD gene mutations

(A) SNVs of ICD genes, **(B)** Circos plot, **(C)** CNVs map

Table S1. Clinical information table for the TCGA training set.

Characteristics	N	%
Age		
< 60	54	31
≥ 60	122	69
Gender		
Female	80	45
Male	97	55
Stage		
Stage I/II	166	96
Stage III/IV	7	4
Grade		
G1	30	17
G2	94	54
G3/G4	50	29
Race		
ASIAN	11	6.4
BLACK OR AFRICAN AMERICAN	6	3.5
WHITE	155	90.1
Histological type		
Pancreas-Adenocarcinoma Ductal Type	146	83.4
Pancreas-Adenocarcinoma-Other Subtype	25	14.3
Pancreas-Colloid (mucinous non-cystic) Carcinoma	4	2.3

Table S2. Clinical information table for the GSE57495 validation set.

Characteristics	N	%
OS. Status		
Deceased	42	67
Alive	21	33
Stage		
Stage I/II	63	100
Histological type		
Tumor	63	100

Table S3. List of ICD genes.

No.	Gene
1	*CD4*
2	*IFNGR1*
3	*FOXP3*
4	*ATG5*
5	*CASP8*
6	*HSP90AA1*
7	*BAX*
8	*P2RX7*
9	*IFNG*
10	*IL17A*
11	*IL1R1*
12	*PIK3CA*
13	*IL1B*
14	*NT5E*
15	*IL6*
16	*IL10*
17	*TLR4*
18	*CASP1*
19	*ENTPD1*
20	*CD8A*
21	*LY96*
22	*NLRP3*
23	*PDIA3*
24	*IFNB1*
25	*EIF2AK3*
26	*CD8B*
27	*MYD88*
28	*IL17RA*
29	*CALR*
30	*PRF1*
31	*CXCR3*
32	*HMGB1*
33	*IFNA1*
34	*TNF*

Table S4. Clinical data for the enrolled patients.

Characteristics	N	%
Age		
≤ 60	17	51.5
≥ 60	16	48.5
Sex		
Female	12	36.4
Male	21	63.6
Smoking		
Never	24	72.7
Ever	9	27.3
Drinking		
Never	31	93.9
Ever	2	6.1
Histologic differentiation		
Low	10	30.3
Moderate and high	23	69.7
Tumor stage		
T1+T2	25	75.8
T3+T4	8	24.2
Nodal stage		
N0	5	15.2
N1-2	28	84.8
Metastatic stage		
MX/M0	32	97.0
M1	1	3.0

Table S5. All primer and siRNA sequences.

Name	Sequence (5'-3')
AJM1-F	CAGCGACTCATACTGTGCGGAT
AJM1-R	GCTCCTCTGTATAGAAGAACTGG
GAPDH-F	ACCACAGTCCATGCCATCAC
GAPDH-R	TCCACCACCCTGTTGCTGTA
siNC-F	UUCUCCGAACGUGUCACGU
siNC-R	ACGUGACACGUUCGGAGAA
siAJM1#1-F	CCGUGUACCAGGACAUCAA
siAJM1#1-R	UUGAUGUCCUGGUACACGG
siAJM1#2-F	GCACCGAGACCAUGUUCAA
siAJM1#2-R	UUGAACAUGGUCUCGGUGC

Table S6. List of 26 genes.

No.	Gene
1	*ADA2*
2	*FYN*
3	*ARRB2*
4	*EBI3*
5	*S1PR2*
6	*RAB8B*
7	*P2RY8*
8	*NOTCH2*
9	*CELF2*
10	*PLA2G2D*
11	*CLEC9A*
12	*GIMAP7*
13	*LIMS1*
14	*GRAP2*
15	*AJM1*
16	*JAM2*
17	*IL10RA*
18	*NLRC3*
19	*MSN*
20	*C1QB*
21	*NRROS*
22	*WDFY4*
23	*CHST11*
24	*SH2B3*
25	*CD1D*
26	*ANKRD44*
